# The Removal of CuO Nanoparticles from Water by Conventional Treatment C/F/S: The Effect of pH and Natural Organic Matter

**DOI:** 10.3390/molecules24050914

**Published:** 2019-03-05

**Authors:** Rizwan Khan, Muhammad Ali Inam, Du Ri Park, Sarfaraz Khan, Muhammad Akram, Ick Tae Yeom

**Affiliations:** 1 Graduate School of Water Resources, Sungkyunkwan University (SKKU) 2066, Suwon 16419, Korea; rizwankhan@skku.edu (R.K.); aliinam@skku.edu (M.A.I.); enfl8709@skku.edu (D.R.P.); 2 Key Laboratory of the Three Gorges Reservoir Region Eco-Environment, State Ministry of Education, Chongqing University, Chongqing 400045, China; Sfk.jadoon@yahoo.com; 3 Shandong Key Laboratory of Water Pollution Control and Resource Reuse, School of Environmental Science and Engineering, Shandong University, Qingdao 266200, China; m.akramsathio@mail.sdu.edu.cn

**Keywords:** agglomeration, coagulation/flocculation/sedimentation, copper oxide nanoparticles, natural organic matter

## Abstract

The increased use of engineered nanoparticles (ENPs), such as copper oxide nanoparticles (CuO NPs), in commercial products and applications raises concern regarding their possible release into freshwater sources. Therefore, their removal from water is important to eliminate adverse environmental and human health effects. In this study, the effects of pH and natural organic matter (NOM), i.e., humic acid (HA) and salicylic acid (SA) on the removal of CuO NPs by coagulation/flocculation/sedimentation (C/F/S) were evaluated. The results indicated that pH significantly affects the coagulation efficiency, where 10–60% CuO NPs removal was achieved under extreme acidic/alkaline conditions. However, at neutral pH, removal of up to 90% was observed with a lower ferric chloride (FC) dosage (0.2 mM). The coagulation efficiency and mechanism were strongly affected by the type of Fe species present in the aqueous phase, which is mainly controlled by pH. Higher concentrations of both HA and SA decrease the CuO NPs agglomeration rate, and thereby improve the colloidal stability due to the NOM molecules adsorbed onto the NPs surface. The presence of hydrophobic HA needs a higher FC dosage of 0.5–0.8 mM than a dosage of hydrophilic SA of 0.25–0.35 mM, to obtain a similar CuO coagulation efficiency. Moreover, higher removals of dissolved organic carbon (DOC) and UV_254_ were observed more in hydrophobic NOM than in hydrophilic. The results of the Fourier transform infrared (FT-IR) analysis of FC composite flocs confirm that the charge neutralization and enmeshment of coagulant might be a possible removal mechanism. The findings of the current study may provide critical information in the prediction of the fate, mobility, and removal of CuO NPs during C/F/S in water treatment.

## 1. Introduction

The usage of metal-based nanomaterials (NMs) in commercial products and applications is increasing rapidly, because of the current advances in nanotechnology. Amongst several NMs, copper oxide nanoparticles (CuO NPs) are widely used in many industries, environmental remediation, agricultural activities, and antimicrobial agents due to their unique specific structural properties [1,2]. The global annual production of CuO NPs was approximately 570 tons/year in 2014 and is predicted to be 1600 tons/year by 2025 [3]. The large production and application of CuO NPs have raised environmental concerns because of their release into the aqueous system, thus increasing the potential risk to human health and aquatic organisms [4].

It has been demonstrated that NPs can attach to the surface of aquatic organisms, such as *Pseudokirchneriella*, *lymphocytes*, and *Fagopyrum esculentum*, and then penetrate into the body of organisms [5]. The CuO NPs can dissociate into Cu^2+^ ions and a high concentration of Cu^2+^ is harmful to both humans and aquatic life. A recent study [6] reported the adverse effect of Cu^2+^ ions on the impaired growth of *Triticum aestivum*, and the increased levels of oxidative stress in microalgae. Long et al. reported the acute toxic effects of NPs on humans, such as damage to cell membranes and DNA structure [7]. The substantial inhibition effect of released Cu^2+^ on the bacterial growth of activated sludge was also well reported, which affected the overall performance of the process [8]. Once released into water, the bioavailability and environmental behavior of NPs largely determine their potential toxicity. Therefore, to reduce the associated ecological risks of NPs, it is essential to understand the dissolution phenomena of CuO NPs in water.

In the natural environment, the colloidal stability of NPs is a crucial parameter to evaluate their fate and mobility. Earlier studies [9,10] reported that various environmental factors, such as NPs properties (particle size, morphology, surface potential) and solution chemistry (pH, ionic strength (IS), natural organic matter (NOM)) might affect the agglomeration and dissolution process of CuO NPs. The surface charge and dissolution of CuO NPs is highly dependent on the media pH, which influences them through protonation/deprotonation reactions on the NPs surface, and thus affects the NPs aggregation behavior in the solution [11]. It has been demonstrated that the divalent ions (Ca^2+^, Mg^2+^) enhance the agglomeration rate of NPs due to the effective compression of the electrical double layer (EDL) [12]. The lower concentration of high molar mass (HMM) hydrophobic NOM, such as humic acid (HA), increase the rate of NPs aggregation, while the higher concentration of HA impedes the NPs aggregation, thus enhancing the colloidal stability of suspension [13]. Bian et al. reported that the HA with a polydentate complexing structure resulted in the enhanced dissolution of ZnO NPs with a pH from 9 to 11 [14]. Moreover, low molar mass (LMM) hydrophilic NOM, such as salicylic acid (SA), may also influence the dissolution of NPs suspension [15]. The coexistence of CuO NPs and NOM in the aquatic system may increase the risks of exposure to aquatic organisms, as well as humans, if the source is being used as potable water.

Coagulation/flocculation/sedimentation (C/F/S) is recognized as the most efficient and economical process to remove colloids and organic and inorganic matter from the water. Previous studies [16,17] reported that multiwall carbon nanotubes (MWCNT) and Cadmium Telluride (CdTe) NPs might be efficiently removed from the aquatic system by alum coagulation. The residual concentration of several ENPs, such as NiO, ZnO, TiO_2,_ and Fe_2_O_3_, by the coagulation process was also reported [18]. Several types of NOM is ubiquitously present in the aquatic environment with concentrations ranging from 0.5 to 10 mg C/L [11]. Moreover, they form complexes with metals and may enhance the mobility and bioavailability of NPs, thus affecting the overall NPs removal in natural waters [12]. In addition, recent studies [16,19] demonstrated the adverse effect of NOM on the coagulant dosage and removal of NPs from water. For example, the decrease in coagulation efficiency in NPs coated NOM was observed due to the electro-steric hindrance effect of sorbed organic molecules [20]. These studies have shown that coagulation seems to be a good option for the removal of CuO NPs from water. However, knowledge of how the characteristics of NOM, such as hydrophilic/hydrophobic characteristics, influence the fate and mobility of CuO NPs in the natural environment is still limited. In addition, earlier studies also seem insufficient regarding the specific effect of hydrophilic/hydrophobic organic substances on the removal behavior of CuO NPs contaminants during the C/F/S process. Therefore, it is imperative to conduct a study that evaluates the influence of water chemistry on the removal of CuO NPs by C/F/S from heterogeneous aqueous environments.

In this study, we investigated the coagulation efficiency of CuO NPs from water under various pH and NOM concentrations. This work also examines the effect of hydrophobic/hydrophilic NOM on the agglomeration, sedimentation, dissolution, and removal of CuO NPs. Furthermore, the obtained data were used to understand the fate, transport, and removal mechanism of CuO NPs in water.

## 2. Materials and Methods

### 2.1. Chemicals Reagents

The CuO NP powder (vendor reported NPs diameter <50 nm), was purchased from Sigma-Aldrich (St. Louis, MO, USA), and used as received (Supplementary Material (SM): Table S1). Two kinds of model natural organic matter (NOM), humic acid (HA) and salicylic acid (SA), were also procured from Sigma-Aldrich. Other chemicals, including hydrochloric acid (HCl), iron (III) chloride hexahydrate (FeCl_3_ 6 H_2_O), and sodium hydroxide (NaOH), were obtained from local suppliers. All stock solutions were prepared in deionized (DI) water produced using a water system (Millipore Co., Bedford, MA, USA).

### 2.2. Preparation of Stock Solutions

The CuO nanopowder (100 mg) was weighed in scintillation vials with a microbalance (Mettler Toledo, Ag Model XP26DR, Mettler Toledo AG, Greidensee, Switzerland) and dispersed into DI water. Before probe sonication, the suspension pH was adjusted to 9 to minimize the dissolution of CuO NPs, and then prob-sonicated with an ultrasonic cell crusher (Bio-Safer 1200-90, Nanjing, China). The effects of sonication power of (100, 600) W and time of (5–30) min on CuO NPs size distribution were evaluated. Afterward, the NPs stock solution was prepared at optimized dispersion parameters. The HA powder (100 mg) was dissolved in 100 mL of DI water, and the solution pH was adjusted to 10 by 0.1 M NaOH to ensure the complete dissolution of HA. The suspension was stirred at 600 rpm overnight to enhance the stability and filtered with a 0.45 µm glass fiber filter, followed by a pH adjustment to 7. The stock solutions of 1 g/L SA and 0.1 M FC were prepared by dissolving the specific amounts of both reagents into the DI water and stored at 4 °C in the dark.

### 2.3. Dissolution and Aggregation Measurements

The dissolution experiments of CuO NPs (10 mg/L) spiked in DI water containing different concentrations of 0–20 mg/L of both NOMs (HA and SA) were carried out in a glass reactor for 24 h at room temperature (RT). After the completion of the experiments, the CuO and Cu^2+^ were separated by centrifuge (Hettich Centrifuger Universal 320-R, Tuttlingen, Germany) at 10,000 rpm for 30 min, and the Cu^2+^ were measured in the suspension. In addition, the agglomeration rate of CuO NPs was measured through the turbidity variation with time, as described in our earlier work [21]. The CuO stock suspension was spiked in water samples containing different concentrations of 0–20 mg/L of both NOMs in the glass vials. To simulate the circulation conditions in natural water bodies, the sample vials were placed on a shaker and shaken at 150 rpm for 24 h. After equilibration, the agglomeration rate of the CuO NPs was measured at different time intervals through a turbidimeter (Hach Benchtop 2100 N, Loveland, CO, USA). Since the turbidity increases with NPs concentration, the agglomeration rate δ(C/C_0_)/δt can be related to the normalized NPs turbidity C/C_0_, where C_0_ and C are the turbidity values at times 0 and t, respectively. The agglomeration rate can be estimated from the initial 5% decrease in normalized turbidity, which tends to occur within the first 1 h for fast aggregation and within 12 h for slow aggregation [22].

### 2.4. Jar-Test Coagulation Experiments

The C/F/S experiments were conducted using a jar tester containing six stirrers (Model: SJ-10, Young Hana Tech Co., Ltd., Gyeongsangbuk-Do, Korea) at 25 °C. Prior to the coagulation experiments, CuO suspension (100 mL) was transferred to a (250 mL) beaker and prob-sonicated. The predetermined dose of FC coagulant of (0.0 to 0.8) mM was dosed, and pH was adjusted with a pH meter (HACH: HQ-40d Portable pH, Multi-Parameter Meter, Thermo Fisher Scientific, Waltham, MA, USA) to a predetermined level, using 0.1 M NaOH and 0.1 M HCl. The coagulation experiments were carried out in the following steps: rapid mixing at 200 rpm for 3 min; subsequently, slower mixing at 40 rpm for 20 min; and settling for 60 min. Peng et al. reported that the solubility of CuO NPs significantly increases at pH below 7 or above 11 [10], thus control experiments within the refereed pH range of 2–11 were selected to evaluate the influence of pH on the dissolution of CuO NPs. The removal efficiency of CuO NPs was evaluated through supernatant turbidity measurements with turbidity meter. Moreover, an aliquot of supernatants of ~30 mL was taken, and centrifuged at 10,000 rpm for 30 min, to determine the concentration of Cu^2+^ ions by Inductively Coupled Plasma Optical Emission Spectroscopy (ICP-OES; Model Varian, Agilent Technologies, Santa Clara, CA, USA).

### 2.5. Other Characterizations and Measurements

The ζ potential of the CuO NPs suspension was evaluated from their determined electrophoretic mobility using Zetasizer (Malvern Nano-ZS, Worcestershire, UK) at (25 ± 1) °C. The particle size distribution of the CuO NPs in suspension was measured with dynamic light scattering (DLS) with a He-Ne laser (λ = 632.8 nm). The residual concentrations of organic matter were determined from UV 254 nm values, which were collected by UV-Vis spectrophotometer (Optizen 2120 UV, Mecasys, Daejeon, Korea). The dissolved organic carbon (DOC) content was determined by a TOC analyzer (TOC-5000A, Shimadzu Corp, Kyoto, Japan). The Fourier Transform Infrared (FT-IR) Spectrum of the CuO-NOM complex and FC flocs was obtained using (FT/IR-4700 JASCO analytical instruments, Easton, PA, USA). The surface composition of the pristine CuO was determined by an X-ray diffractometer (Rigaku D-max C III Corporation, Tokyo, Japan) and Raman spectroscopy with high-resolution confocal Lab Ram HR Evolution Horiba Jobin Yvon, Bensheim, Germany.

## 3. Results

### 3.1. Characterization of CuO NPs

**Figure 1:**
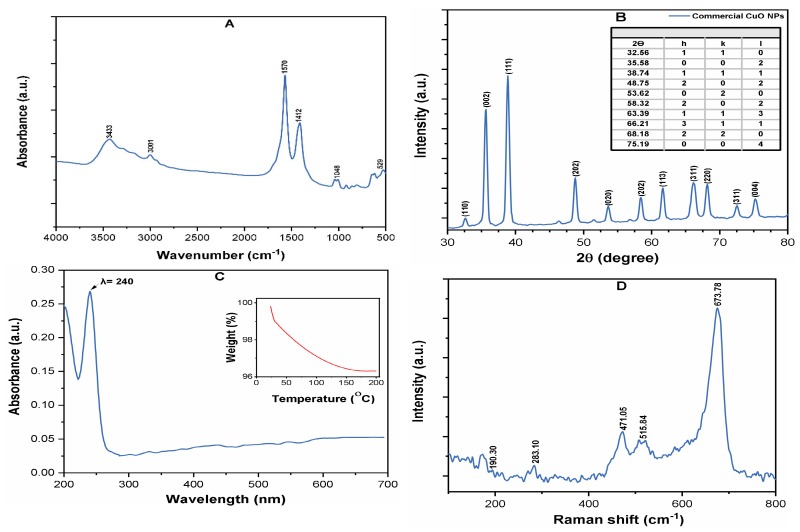
The characterization: (**A**) Fourier Transform Infrared (FT-IR) spectra; (**B**) X-ray diffraction (XRD) survey spectrum; (**C**) UV-Vis spectra of CuO nanoparticles (NPs) (10 mg/L) in deionized (DI) water at pH 7, where the inset shows the Thermogravimetric analysis (TGA) (%) purity; (**D**) Raman spectrum of CuO powder.

Table S1 of the SM indicates some important physicochemical properties of the CuO NPs. Figure 1A depicts the FT-IR spectrum of commercial CuO NPs, which shows the peaks at 529 cm^−1^, corresponding to the Cu–O stretching vibrations [23]. The effect of the sonication power of 100–600 W and time of 5–30 min on the CuO NPs size distribution in suspension were investigated by DLS measurements. The best dispersion was obtained at a sonication power of 400 W and time of 25 min; however, upon further increase of both operating parameters, no substantial variation in the particle size was observed (Figure S1A of the SM).

The hydrodynamic diameter (HDD) of the CuO NPs suspension was measured as 225  ±  27 nm by DLS at pH 7.0 (Figure S1B of the SM), thus indicating a much larger size of NPs in DI water, as compared to the vendor reported size (<50 nm). This is likely related to the increasing Van der Waals (vdW) attraction or electrostatic forces between the NPs, which led to the formation of larger aggregates in aqueous solution [22]. The crystalline structure of CuO NPs is shown in the XRD spectrum (Figure 1B). The observed diffraction peaks at 32.56, 35.58, 38.74, 48.75, 53.62, 58.32, 63.39, 66.21, 68.18, and 75.19 units correspond to the (110), (002), (111), (202), (020), (202), (113), (311), (220), (311) and (004) planes, confirming the high crystallinity of CuO NPs [24]. The presence of CuO NP was also evidenced by the UV-Vis spectra, which showed a strong excitation of the surface plasmon resonance (SPR) peak at a wavelength of 240 nm (Figure 1C). Figure 1D shows the Raman spectra of pristine CuO NPs, where the wavenumbers were observed around (283, 515, and 619) cm^−1^, all of which belong to the Ag and Bg modes of CuO. A weak characteristic peak at about 190 cm^−1^ for the Cu–O structures was observed in the Raman spectrum [25].

### 3.2. The Effect of pH on the Dissolution and Surface Properties of CuO NPs

**Figure 2:**
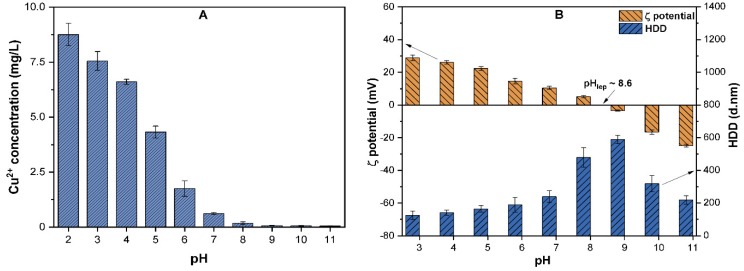
(**A**) The released Cu^2+^ ions (mg/L); (**B**) ζ potential and hydrodynamic diameter (HDD) of CuO NPs (10 mg/L) as a function of suspension pH (3–11).

The pH of the aqueous solution may affect the NPs properties, such as their size, solubility, and surface charge. The effects of various pH values (2–11) on the solubility, ζ potential, and HDD of CuO NPs were investigated (Figure 2). The Cu^2+^ ions released from CuO (10 mg/L) at pH ranging (2–5) was up to 4.3–8.5% of the total Cu. However, above pH 6, the solubility of CuO NPs declined, and at pH 7, less than 0.6% of the total Cu was observed (Figure 2A). These results are in good agreement with those reported by Peng et al., who observed an inverse relationship between the suspension pH and solubility of CuO [10]. Moreover, at highly acidic conditions, the H^+^ ions react with CuO NPs and release a substantial amount of Cu^2+^ ions, while at alkaline conditions, the high concentration of OH^−^ may induce the formation of Cu (OH)^+^ complexes [26]. As shown in Figure 2B, the variation in the solution pH also affects the surface potential and HDD of CuO NPs. The ζ potential was positive when the pH ranged between 3.0 to ~8.5 and remained negative at alkaline pH values of 9.0–11.0. The Isoelectric point pH_iep_ of CuO NPs was observed at/near pH 8.6, where the ζ potential of −2.3 ± 2.4 mV and HDD of above 590 ± 45 nm were found to be the highest among all the tested pH values. These results are in agreement with the results of previous studies [10,12,23] on CuO, which determined the pH_iep_ of CuO to be between ~8.2 and 8.8. The previous study [10] observations also affirm our result that the ζ potential value is positive when pH < pH_iep_, and becomes negative at pH > pH_iep_. Moreover, particles with a ζ potential below ±15 mV are assumed to be unstable without any hindrance and tend to aggregate, while NPs with ζ potential above ±30 mV are considered to be well-stabilized [27]. At pH_iep_, high electrostatic interaction reduces the repulsion between NPs; thus each collision causes adherence and enhances the HDD of particles.

In our study, before pH_iep_, the ζ potential remains positive ((25 ± 1.741) to (5.14 ± 1.5)) for the pH values 3.0–8.0 and HDD ranges between 160 ± 35 and ~480 ± 59 nm. In addition, an increase in the pH results in NPs stabilization due to an increase in the concentration of hydroxyl radical (OH–) and electrostatic repulsion between the NPs. In contrast, at higher alkaline pH values, the vdW forces weaken, thus the HDD of the particle decreases [21].

#### Effect of pH on CuO NPs Removal

**Figure 3:**
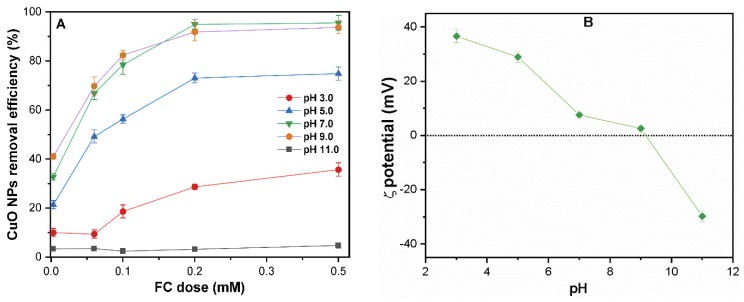
(**A**)The effect of pH on the removal of CuO NPs (10 mg/L), and (**B**) ζ potential of CuO NPs suspensions at 0.2 mM FC dose under pH range (3 to 11).

Figure 3A presents the coagulation efficiency of CuO NPs (10 mg/L) under various pH ranges of 3–11 and an FC dosage of 0–0.5 mM. The pH-dependent coagulation was observed over the entire pH range (3–11), and more than 70% NPs removal was achieved at FC dosage (0.2 mM), except under highly acidic/alkaline conditions. A similar effect of pH on the removal of CuO without coagulant was observed, as shown in Figure S2A of the SM. The removal of CuO NPs drastically decreases at pH values of 3 and 11, where less than 40% of CuO were removed, even at higher FC dosage (0.5 mM). The ζ potential of NPs in the presence of FC (0.2 mM) decreases from 36 to −28 mV as pH increases from 3 to 11 (Figure 3B). Thus, variation in the ζ potential of colloids with pH is likely to play a significant role in determining the coagulation efficiency of CuO NPs by C/F/S.

It is noteworthy that the variation in pH also affects the solubility and speciation of the Fe species in aqueous solution (Figure S2B of the SM). In the studied pH (3–11), the dominant species include one or more Fe^+3^, Fe(OH)^2+^, Fe(OH)_2_^+^, Fe(OH)_3_, and Fe(OH)_4_^−^ complexes in solution. The variation of Fe species in the aqueous phase profoundly influenced its interaction with CuO NPs and thereby determines their coagulation efficiency. The charge neutralization, EDL compression, and enmeshment of coagulant may be the possible driving mechanisms for FC-induced coagulation. Like other ENPs (i.e., CNTs, C60, and ZnO), the absolute surface potential and electrostatic repulsive forces might hinder the aggregation of CuO NPs in suspension [20,28]. However, pH might influence the coagulation mechanism of CuO NPs due to the change in NPs surface potential and Fe speciation with pH.

At a pH of 9, as the pH_iep_ value of CuO NPs is ~8.6, the oxygen-containing groups at the surface of the CuO NPs are mostly deprotonated, which might enhance the CuO NPs agglomeration [23]. With the increased amount of Fe(III) species (Fe(OH)_2_⁺, Fe(OH)_3_, and Fe(OH)_4_^−^), EDL compression occurs, which may lead to effective CuO removal [16]. Moreover, under a neutral pH condition, the interaction behavior between Fe(III) species (Fe(OH)_2_⁺) and CuO NPs increases (Figure S2B of the SM); therefore, a low FC dose presents higher coagulation efficiency [29]. The Fe(III)-induced cation-bridging effect on colloids becomes dominant at the optimum dosage (0.2 mM), which resulted in the enhanced coagulation of CuO NPs. Moreover, based on the speciation of Fe (III), a large amount of Fe(OH)3 precipitates are generated by an FC coagulation at pH 7; thus these precipitates serve as nucleation sites for entrapping more NPs colloids and leads to NPs agglomeration [30]. At pH (7 and 5), the most dominant Fe(III) species in suspension are Fe(OH)_2_⁺, Fe(OH)^2+^, and Fe(OH)_3_, respectively; consequently, the enmeshment of NPs may result in the flocculation of CuO colloids [31]. Moreover, the ζ potential of the CuO-FC mixture decreases up to ~9 mV due to the combination of cation-bridging and EDL compression, resulting in CuO NPs coagulation. At a pH of 11, the oxygen functional groups on the NPs are mostly deprotonated, and CuO is stable in solution, due to substantial negative charges. Meanwhile, Fe(III) is mostly present in suspension as Fe(OH)_4_^−^, which is also negatively charged. Therefore, electrostatic repulsion between the CuO and Fe species is maximum at this pH. The principal phenomenon for coagulation at a higher pH might be nucleation due to the minimal interactions between CuO and FC [32].

### 3.3. The Effect of the FC Dose on the Removal of CuO NP

**Figure 4:**
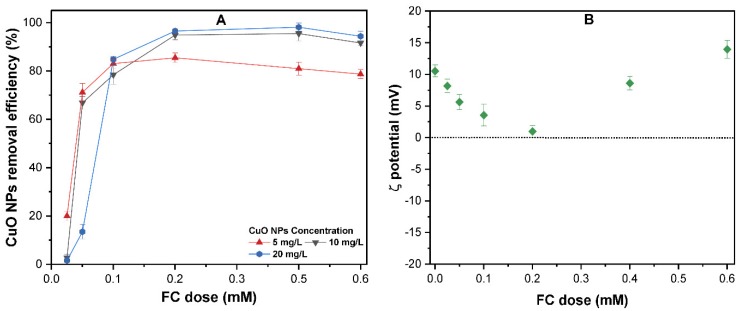
(**A**)The effect of various ferric chloride (FC) dosages of 0–0.6 mM on the coagulation removal of different concentrations of CuO NPs of 5–20 mg/L; (**B**) ζ potential of CuO NPs (10 mg/L) suspensions at various FC dosages at pH = 7.

Considering the dissolution of CuO NPs in acidic conditions and the simulation of the solution chemistry of real water treatment, further experiments were conducted with various FC dosages of 0.025–0.6 mM and CuO NPs concentration of 5–20 mg/L at pH 7. As shown in Figure 4A, the removal efficiency is enhanced with an increase in CuO NPs concentration, and more than 80% of CuO NPs of 5–20 mg/L were effectively removed at an FC dosage above 0.2 mM. A similar observation has been reported in previous studies [33,34], which reported a higher removal of 60–80% of C60 and pristine CNTs with alum and polyaluminum chloride (PAC) coagulants. The increase in FC dosage above 0.2 mM caused an insignificant effect on the CuO NPs removal. Interestingly, the removal efficiency of CuO NPs with FC is unlike the other ENPs, such as Ag NPs or TiO_2_ [35,36], where the removal curves of NPs showed an inverted “U” type pattern with increasing coagulant dosage. Initially, the charge neutralization of CuO by FC results in enhanced removal, followed by a decrease in removal at a higher dosage due to the presence of more positive Fe(III) species in suspension. The effect of FC dosage on the surface charge of CuO NPs was measured, as shown in Figure 4B. The ζ potential of CuO decreased significantly at a lower FC dose (below 0.2 mM), approached zero at 0.2 mM FC, and then increased to 15 ± 1.284 mV at FC dosage 0.6 mM. As mentioned earlier, the dominant form of Fe(III) present in neutral pH is Fe(OH)_2_⁺. At higher concentrations of these Fe species, the primary mechanism of CuO coagulation should be enmeshment, primarily by Fe(OH)_2_⁺ [37].

### 3.4. The Effect of NOM on the Dissolution and Aggregation of CuO NP

The interaction of released NPs with organic pollutants in the aquatic environment may affect the dissolution and, consequently, increase their bioavailability to the organism [15]. Therefore, processes such as aggregation may control the NPs associated potential risks in an aqueous system. The release of Cu^2+^ and the change in the suspension pH over time were observed at various concentrations of 0–20 mg/L of both NOMs. As presented in Figure 2A, in the absence of NOM, the measured concentration of ion at pH 7 was only 0.6 mg/L; however, the concentration increased gradually with increasing NOM concentration. At higher concentration of HA and SA (20 mg/L) the measured concertation of Cu^2+^ was found to be around 2.36 ± 0.03 and 1.54 ± 0.01 mg/L, respectively (Table S2 of the SM). This is in agreement with previous work showing the strong complexation and coordination ability of hydrophobic NOM with metal ions [12]. On the other hand, the complex molecule may absorb onto the flat terraces, and polarize the metal–oxygen bonds of the lattice surface. Therefore, a higher concentration of HA forms additional dissolution hot spots on the flat terraces than in the edges, resulting in the enhanced dissolution of CuO NPs [38]. In contrast, the lower dissolution in the SA containing sample may be related to NOM-coated NPs, which serve as a competitive sink to form oxidation compounds, hence hindering the oxidation of CuO NPs [11]. Moreover, it is postulated that the aggregation of NPs suspension reduced the available NPs reactive surfaces in the aqueous phase. Thus, aggregation may play an essential role in the CuO NPs dissolution in a heterogeneous system.

**Figure 5:**
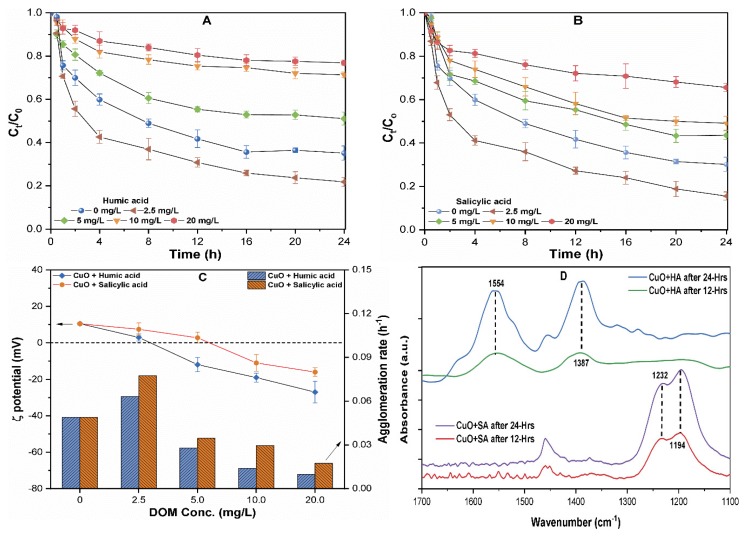
The effect of various concentrations of 0–20 mg/L of (**A**) humic acid (HA), and (**B**) salicylic acid (SA) on the aggregation kinetics of CuO NPs; (**C**) ζ potential and agglomeration rate of CuO NPs (10 mg/L) at different concentrations of NOM; (**D**) FT-IR spectrum of CuO-NOM complexes at pH = 7.

Figure 5 shows the results of the agglomeration behavior of CuO (10 mg/L) NPs dispersed in various concentrations of HA and SA of 0–20 mg/L containing waters. It can be observed that a 40% loss of turbidity occurred in the initial hours for CuO NPs suspended in waters containing both NOMs (2.5 mg/L) (Figure 5A,B). The ζ potential of CuO NPs in these suspensions was found near the pH_iep_ (Figure 5C); thus, the increased agglomeration rate might be attributable to charge neutralization between the positively charged NPs and negatively charged NOM groups [14]. Moreover, electrostatic attraction forces between the CuO NPs and negatively charged groups of NOM increased near/at pH_iep_, resulting in the enhanced aggregation of NPs in solution [18]. However, only a 10–25% loss of solution turbidity within the first hour with a smaller agglomeration rate at a higher concentration of 10–20 mg/L of HA and SA was observed (Figure 5A,B).

The coating of organic molecules on the surface of NPs might alter the surface potential, as well as increase the steric hindrance effect among the NPs [22]. The attachment of negatively charged carboxylates and phenolics resulted in the charge reversal (positive to negative) of CuO NPs (Figure 5C). Figure 5C shows that the suspension containing a higher concentration of hydrophobic HA presented the slowest rate of agglomeration of 0.01–0.06 h^−1^ in comparison with the same concentration of 0.02–0.08 h^−1^ of hydrophilic SA. Similar observations were earlier reported by Zhu et al., who reported that the phenolic and carboxylate groups in hydrophobic NOM might adsorb on the NPs surface, and thus enhance the colloidal stability by inverting the ζ potential of the TiO_2_ NPs solution [39,40]. The organic anions in HA significantly affect the charge density and structure of the surface adsorbed layer, thereby affecting the NPs interaction during the aggregation process [23]. These results suggest that the type of NOM affects the coating mechanism, thus affecting the ζ potential and stability of CuO NP in suspension.

In addition, the FT-IR spectra of CuO NP-complexes with both NOMs (HA, SA) after 12 and 24 h of interaction were analyzed for the possible adsorption of functional groups onto the CuO NPs surface. In comparison with the pristine CuO NPs, CuO–HA complexes show an increase in the peak intensities with a time at 1554 and 1387 cm^−1^, which are ascribed to the stretching vibration of ketones (C=O) and phenols, respectively [41]. This broadening in peak intensities might be related to the presence of various functional groups in HA that may form polar co-ordinations to the NPs surface. The peaks in the CuO-SA complexes around 1232 and 1194 cm^−1^ correspond to the enrichment of carbohydrate-OH and aliphatic groups [15]. Therefore, the appearance of new peaks in the CuO–NOM complex confirms the attachment of oxygen-containing functional groups onto the CuO NPs surface. The functional groups present in HA and SA, such as phenolic (–ArO–), carboxylic (–COO–), and amine, have strong potential for chelation with metal ions [22]. Therefore, the inner sphere metal complexion might provide additional crystal lattices and outermost surface defects to the NOM molecules, and promote the dissolution of CuO NPs. From an environmental fate and risk assessment perspective, the results imply that CuO NPs may dissolve rapidly in the presence of a higher NOM concentration, thus increasing toxicity in the vicinity of the released water body. However, low concentrations of NOM decrease the stability of NPs, hence limiting their mobility.

### 3.5. The Effect of NOM on CuO NPs Removal

**Figure 6:**
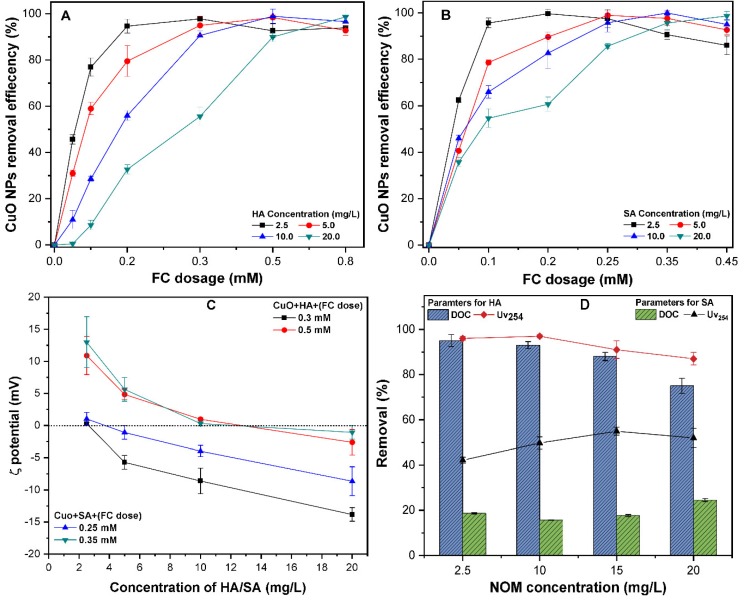
Effect of various concentrations of 0–20 mg/L of (**A**) HA, and (**B**) SA, on the removal efficiency of CuO NPs (10 mg/L) at a pH of 7.0; (**C**) ζ potential of CuO NPs (10 mg/L) at various FC dosages; (**D**) the removal of DOC and UV_254_ from natural organic matter (NOM) samples at optimum FC dosage.

The different NOMs present in subsurface and groundwater environments might adsorb onto the surface of the NPs and affect their transportation behavior. Figure 6 shows the coagulation efficiency of CuO (10 mg/L) in the presence of HA and SA of 0–20 mg/L at a neutral pH of 7.0. It was observed that at a lower FC dosage below 0.2 mM, the presence of HA remarkably decreases the coagulation of CuO NPs (Figure 6A). For example, at an FC dosage of 0.1 mM, the coagulation efficiency of CuO was found to be between 5–20% in the presence of a higher concentration of (10–20 mg/L) HA. Moreover, the waters containing SA require a lower FC dosage to achieve a higher removal of CuO NPs (Figure 2B). A similar observation has been reported in the literature, where water with hydrophobic NOM requires a higher coagulant dose to obtain a higher removal [35]. With increasing NOM concentrations of 10–20 mg/L, the ζ potential of CuO in the presence of FC remain negative due to the adsorption of negatively charge NOM molecules (Figure 6C). The higher concentration of both NOMs improves the colloidal stability due to the electro steric repulsion. The previous studies [16,17,21] on other ENPs, such as CdTe, C60, and ZnO NPs, reported that the surface coating of NOMs onto NPs resulted in charge reversal (positive to negative) and a more coagulant dosage being required to destabilize the suspension.

These results show that CuO NPs removal depends upon the characteristics of the NOM present in the source water. The removal efficiency of CuO at FC dosage of 0.35 and 0.5 mM increases up to 99% in the presence of SA and HA, respectively. Moreover, the excess coagulant dosage promotes the re-stabilization of the CuO suspension, and thereby reduces the NPs removal. This phenomenon is consistent with previous studies [21,35,42] that state that the oversaturation of polyelectrolytes might result in charge inversion and the restabilization of coagulated colloids in solution. At an optimum FC dosage, the NOM molecules and adsorption of negative charges onto the NPs surface might bind with the Fe(III) ions via electron coupling interaction, thus forming CuO–Fe–NOM complexes. It has been reported that charged Fe species have a strong binding affinity for NPs and NOM molecules [43]. Moreover, the Fe ions could serve as a bridging agent between the surface of the NPs and NOMs molecules (Figure S2B of the SM), and thus significantly enhance the C/F/S process. Similar observations were reported, where cations decrease the colloidal stability of NPs, through the bridging with various functional groups of HA [20,44].

The removal of NOM-related parameters, DOC and UV_254_ at the optimum dose (OD) of FC was measured (Figure 6D). The results show that at OD, the lowest removal of DOC and UV_254_ (40%) in SA-containing suspensions and the highest (95%) in HA was obtained (Figure 6D). This revealed that the NOM hydrophobic fraction might be removed more efficiently in comparison to the hydrophilic fraction. These results are in good agreement with earlier studies [35,45] and can be described as follows: for the solution with humic substance and HMM aromatic compounds, more than 60% of DOC removal is achieved because the coagulation process is primarily controlled by NOM. Likewise, for waters containing NOM composed of non-humic and LMM compounds, NOM has little influence on coagulation, thus only 30% of DOC was removed. Interestingly, irrespective of the NOM type, more UV_254_ is reduced than DOC, which indicates that aromatic compounds are removed more easily than other NOM fractions. This phenomenon might be attributed to the low pH_iep_ of hydrophobic NOM because of the higher content of phenolic and carboxylic groups, thus resulting in higher adsorption onto the NPs surface, as reported in an earlier study [35]. Moreover, higher anionic binding sites in HA facilitate effective charge neutralization with cationic Fe(III) hydrolysis products than the hydrophilic NOM at the optimum dosage [41]. The DOC and UV_254_ removal in hydrophilic NOM solutions were 25 and 60%, respectively (Figure 6D). This may be ascribed to the presence of LMM compounds and weaker acidic groups in these waters, hence hindering the competitive interactions between NOM molecules and coagulants [46]. These results are consistent with an earlier study [47], which has shown the low removal of hydrophilic NOM fraction from water by coagulation. This indicates that the NOM type may affect the coagulation efficiency and applied coagulant dosage. Moreover, the hydrophilic NOM fraction required post-treatment because of its lower removal during the conventional C/F/S process. It is noteworthy that the removal efficiency of NPs might vary with other interfering ions and physicochemical properties of natural waters. Based on our results, the characteristic of NOM appears to be a vital factor affecting the treatment efficiency and should be considered in any mechanistic approach to water treatment.

### 3.6. The Mechanism of CuO NPs Removal by C/F/S

**Figure 7:**
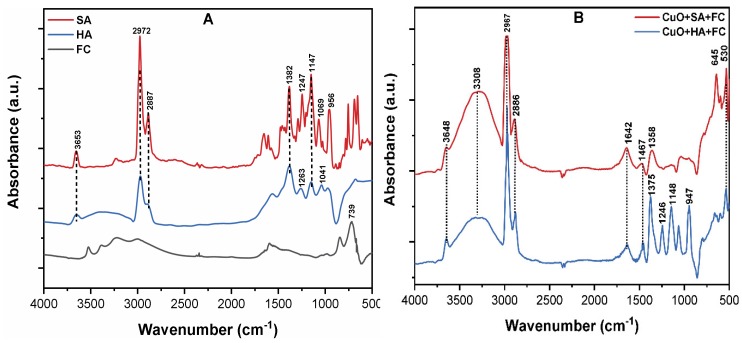
(**A**) The FT-IR spectra of the pristine SA, HA, and FC; (**B**) CuO-HA, SA, and FC flocs obtained after the C/F/S process.

Figure 7A shows that the FT-IR spectrums of NOM, FC, and CuO-NOMs flocs by FC were analyzed to explain the possible removal mechanism. The peaks at 3653, 2972, and 2887 cm^−1^ are attributable to O–H and the symmetric and asymmetric stretching vibrations of aliphatic C–H and C–H_2_, respectively [41]. The peaks appearing at 1382, 1398, and 1263 cm^−1^ are associated with the symmetric and anti-symmetric stretching vibration of CH_3_, COO−, and C–O, respectively [48]. In addition, the few peaks observed at 1263, 1147, 1041, 1069, 956, and 739 cm^−1^ are ascribed to the stretching vibration of C–OH, C–O, C–O–C, groups, and F–O bond, respectively [20,49].

Figure 7B shows the IR spectrum of CuO–NOM flocs by FC. The broad peak in both flocs in the range 3648–2886 cm^−1^ is due to the hydrolyzed products and polar interactions of Fe(III) ions with contaminants, which leads to the formation of metal complexes [46]. The significant shift in the peak of C=O in ketones from 1263 to 1246 cm^−1^ further confirms the involvement of aromatic esters and carboxylic groups in complex coordination with metal ions during the C/F/S process [20,29]. Moreover, the shift in peak from 1041 to 1148 cm^−1^ indicates the stretching vibrations of Fe–O/Cu–O bonds. The bands that appear at 947 and 645 cm^−1^ are ascribed to the bending vibration of Cu–O–Fe and Fe–O–Cu/Fe–OH–Cu bonds [32,44]. Therefore, the significant shifts in distinct peaks in the FC composite flocs suggest the strong complex reaction of colloids with FC. Therefore, it can be inferred that during the coagulation process, complexes of different compositions were formed in CuO NPs suspension containing NOM. The principal mechanism for CuO NPs with NOM might be the combination of entrapment, charge neutralization, metal complexation, and ligand exchange adsorption. However, because of the different characteristics of NOM, the removal mechanism may vary according to the specific type of organic substances present in the source water.

## 4. Conclusions

This study showed that C/F/S is an effective process to remove CuO NPs from aqueous environments. However, the coagulation efficiency of CuO depends on the applied coagulant dosage and water chemistry, i.e., pH and characteristics of NOM. When the pH ranges between 7 and 9, the coagulation efficiency of CuO at 0.2 mM FC was found to be higher than 95%; however, it remained below 40% in highly acidic and alkaline pH conditions. The higher concentration of HA and SA in aqueous suspension results in a distinctly adverse effect on the agglomeration of CuO NPs, while the effect of hydrophobic ligands was more pronounced than that of the hydrophilic ligands. The FT-IR analysis of CuO-NOM composite indicated the increase in IR intensities over time due to the enhanced adsorption of NOM molecules onto the NPs surface. Moreover, the result showed that the NOM characteristics significantly affect the optimum FC dosage for the CuO NPs removal from aqueous solution. The higher CuO NPs removal was obtained at a lower FC dosage in hydrophilic suspension in comparison to that in the hydrophobic suspension. Moreover, the highest removals of up to 95% in DOC and UV_254nm_ were achieved in the hydrophobic NOM, in comparison to 40% in the hydrophilic NOM. The mechanisms, such as entrapment, charge neutralization, ligand exchange adsorption, and metal complexation, might play important roles in the removal of CuO NPs composite pollutant by C/F/S. This study elucidates that the characteristic of organic substance might influence the fate, mobility, and coagulation efficiency of NPs in water/wastewater treatment processes.

## Supplementary Materials

The following are available online at https://www.mdpi.com/1420-3049/24/5/914/s1, Figure S1: (A) Effects of sonication time of 5–30 min and power of 100–600 W on the dispersion stability of CuO NPs stock (100 mg/L) in DI water; (B) Size distribution by intensity of CuO NPs in DI water after 30 min sonication with ultrasonic power of 400 W; Figure S2: (A) Removal efficiency of CuO NPs (10 mg/L) under control condition at various pH values; (B) calculated solubility and speciation of F(III) as a function of solution pH; Table S1: Physicochemical properties of CuO NPs used in the current study; Table S2: Dissolution of CuO NPs and the change of suspension pH.

## References

[1] Wang Z.L. (2004). Zinc oxide nanostructures: Growth, properties and applications. J. Phys. Condens. Matter.

[2] Cioffi N., Ditaranto N., Torsi L., Picca R.A., Sabbatini L., Valentini A., Novello L., Tantillo G., Bleve-Zacheo T., Zambonin P.G. (2005). Analytical characterization of bioactive fluoropolymer ultra-thin coatings modified by copper nanoparticles. Anal. Bioanal. Chem..

[3] Keller A.A., McFerran S., Lazareva A., Suh S. (2013). Global life cycle releases of engineered nanomaterials. J. Nanoparticle Res..

[4] Handy R.D., Von Der Kammer F., Lead J.R., Hassellöv M., Owen R., Crane M. (2008). The ecotoxicology and chemistry of manufactured nanoparticles. Ecotoxicology.

[5] Nel A., Xia T., Mädler L., Li N. (2006). Toxic potential of materials at the nanolevel. Science.

[6] Aruoja V., Dubourguier H.-C., Kasemets K., Kahru A. (2009). Toxicity of nanoparticles of CuO, ZnO and TiO_2_ to microalgae Pseudokirchneriella subcapitata. Sci. Total Environ..

[7] Long T.C., Saleh N., Tilton R.D., Lowry G.V., Veronesi B. (2006). Titanium dioxide (P25) produces reactive oxygen species in immortalized brain microglia (BV2): Implications for nanoparticle neurotoxicity. Environ. Sci. Technol..

[8] Hou J., Miao L., Wang C., Wang P., Ao Y., Lv B. (2015). Effect of CuO nanoparticles on the production and composition of extracellular polymeric substances and physicochemical stability of activated sludge flocs. Bioresour. Technol..

[9] Aiken G.R., Hsu-Kim H., Ryan J.N. (2011). Influence of Dissolved Organic Matter on the Environmental Fate of Metals, Nanoparticles, and Colloids.

[10] Peng C., Shen C., Zheng S., Yang W., Hu H., Liu J., Shi J. (2017). Transformation of CuO Nanoparticles in the Aquatic Environment: Influence of pH, Electrolytes and Natural Organic Matter. Nanomaterials.

[11] Philippe A., Schaumann G.E. (2014). Interactions of dissolved organic matter with natural and engineered inorganic colloids: A review. Environ. Sci. Technol..

[12] Son J., Vavra J., Forbes V.E. (2015). Effects of water quality parameters on agglomeration and dissolution of copper oxide nanoparticles (CuO-NPs) using a central composite circumscribed design. Sci. Total Environ..

[13] Grillo R., Rosa A.H., Fraceto L.F. (2015). Engineered nanoparticles and organic matter: A review of the state-of-the-art. Chemosphere.

[14] Bian S.W., Mudunkotuwa I.A., Rupasinghe T., Grassian V.H. (2011). Aggregation and dissolution of 4 nm ZnO nanoparticles in aqueous environments: Influence of pH, ionic strength, size, and adsorption of humic acid. Langmuir.

[15] Khan R., Inam M., Zam S., Park D., Yeom I. (2018). Assessment of Key Environmental Factors Influencing the Sedimentation and Aggregation Behavior of Zinc Oxide Nanoparticles in Aquatic Environment. Water.

[16] Zhang Y., Chen Y., Westerhoff P., Crittenden J.C. (2007). Stability and removal of water soluble CdTe quantum dots in water. Environ. Sci. Technol..

[17] Hyung H., Kim J.H. (2009). Dispersion of C60 in natural water and removal by conventional drinking water treatment processes. Water Res..

[18] Zhang Y., Chen Y., Westerhoff P., Hristovski K., Crittenden J.C. (2008). Stability of commercial metal oxide nanoparticles in water. Water Res..

[19] Wang H.T., Ye Y.Y., Qi J., Li F.T., Tang Y.L. (2013). Removal of titanium dioxide nanoparticles by coagulation: Effects of coagulants, typical ions, alkalinity and natural organic matters. Water Sci. Technol..

[20] Khan R., Inam M., Park D., Zam Zam S., Shin S., Khan S., Akram M., Yeom I. (2018). Influence of Organic Ligands on the Colloidal Stability and Removal of ZnO Nanoparticles from Synthetic Waters by Coagulation. Processes.

[21] Khan R., Inam M., Iqbal M., Shoaib M., Park D., Lee K., Shin S., Khan S., Yeom I. (2019). Removal of ZnO Nanoparticles from Natural Waters by Coagulation-Flocculation Process: Influence of Surfactant Type on Aggregation, Dissolution and Colloidal Stability. Sustainability.

[22] Keller A.A., Wang H., Zhou D., Lenihan H.S., Cherr G., Cardinale B.J., Miller R., Zhaoxia J.I. (2010). Stability and aggregation of metal oxide nanoparticles in natural aqueous matrices. Environ. Sci. Technol..

[23] Miao L., Wang C., Hou J., Wang P., Ao Y., Li Y., Lv B., Yang Y., You G., Xu Y. (2016). Effect of alginate on the aggregation kinetics of copper oxide nanoparticles (CuO NPs): Bridging interaction and hetero-aggregation induced by Ca^2+^. Environ. Sci. Pollut. Res..

[24] Selvarajan E., Mohanasrinivasan V. (2013). Biosynthesis and characterization of ZnO nanoparticles using Lactobacillus plantarum VITES07. Mater. Lett..

[25] Deng Y., Handoko A.D., Du Y., Xi S., Yeo B.S. (2016). In situ Raman spectroscopy of copper and copper oxide surfaces during electrochemical oxygen evolution reaction: Identification of CuIII oxides as catalytically active species. ACS Catal..

[26] Miao A.J., Zhang X.Y., Luo Z., Chen C.S., Chin W.C., Santschi P.H., Quigg A. (2010). Zinc oxide-engineered nanoparticles: Dissolution and toxicity to marine phytoplankton. Environ. Toxicol. Chem..

[27] Zhou D., Keller A.A. (2010). Role of morphology in the aggregation kinetics of ZnO nanoparticles. Water Res..

[28] Adeleye A.S., Keller A.A. (2014). Long-term colloidal stability and metal leaching of single wall carbon nanotubes: Effect of temperature and extracellular polymeric substances. Water Res..

[29] Yang Z.L., Gao B.Y., Yue Q.Y., Wang Y. (2010). Effect of pH on the coagulation performance of Al-based coagulants and residual aluminum speciation during the treatment of humic acid–kaolin synthetic water. J. Hazard. Mater..

[30] Popowich A., Zhang Q., Le X.C. (2015). Removal of nanoparticles by coagulation. J. Environ. Sci..

[31] Abbott Chalew T.E., Ajmani G.S., Huang H., Schwab K.J. (2013). Evaluating nanoparticle breakthrough during drinking water treatment. Environ. Health Perspect..

[32] Wang Y., Xue N., Chu Y., Sun Y., Yan H., Han Q. (2015). CuO nanoparticle–humic acid (CuONP–HA) composite contaminant removal by coagulation/ultrafiltration process: The application of sodium alginate as coagulant aid. Desalination.

[33] Ma S., Liu C., Yang K., Lin D. (2012). Coagulation removal of humic acid-stabilized carbon nanotubes from water by PACl: Influences of hydraulic condition and water chemistry. Sci. Total Environ..

[34] Sousa V.S., Corniciuc C., Ribau Teixeira M. (2017). The effect of TiO_2_ nanoparticles removal on drinking water quality produced by conventional treatment C/F/S. Water Res..

[35] Sun Q., Li Y., Tang T., Yuan Z., Yu C.-P. (2013). Removal of silver nanoparticles by coagulation processes. J. Hazard. Mater..

[36] Kosmulski M. (2009). Compilation of PZC and IEP of sparingly soluble metal oxides and hydroxides from literature. Adv. Colloid Interface Sci..

[37] Odzak N., Kistler D., Behra R., Sigg L. (2014). Dissolution of metal and metal oxide nanoparticles in aqueous media. Environ. Pollut..

[38] Zhu M., Wang H., Keller A.A., Wang T., Li F. (2014). The effect of humic acid on the aggregation of titanium dioxide nanoparticles under different pH and ionic strengths. Sci. Total Environ..

[39] Giasuddin A.B.M., Kanel S.R., Choi H. (2007). Adsorption of humic acid onto nanoscale zerovalent iron and its effect on arsenic removal. Environ. Sci. Technol..

[40] Barathi M., Kumar A.S.K., Kumar C.U., Rajesh N. (2014). Graphene oxide–aluminium oxyhydroxide interaction and its application for the effective adsorption of fluoride. RSC Adv..

[41] Chowdhury I., Duch M.C., Mansukhani N.D., Hersam M.C., Bouchard D. (2013). Colloidal properties and stability of graphene oxide nanomaterials in the aquatic environment. Environ. Sci. Technol..

[42] Edzwald J.K., Tobiason J.E. (1999). Enhanced coagulation: US requirements and a broader view. Water Sci. Technol..

[43] Sharp E.L., Jarvis P., Parsons S.A., Jefferson B. (2006). Impact of fractional character on the coagulation of NOM. Colloids Surf. A Physicochem. Eng. Asp..

[44] Sharp E.L., Parson S.A., Jefferson B. (2006). Coagulation of NOM: Linking character to treatment. Water Sci. Technol..

[45] Alberts J.J., Filip Z. (1998). Metal binding in estuarine humic and fulvic acids: Ftir analysis of humic acid-metal complexes. Environ. Technol..

[46] Inam M.A., Khan R., Park D.R., Ali B.A., Uddin A., Yeom I.T. (2018). Influence of pH and Contaminant Redox Form on the Competitive Removal of Arsenic and Antimony from Aqueous Media by Coagulation. Minerals.

[47] Wang H., Zhao X., Han X., Tang Z., Song F., Zhang S., Zhu Y., Guo W., He Z., Guo Q. (2018). Colloidal stability of Fe_3_O_4_ magnetic nanoparticles differentially impacted by dissolved organic matter and cations in synthetic and naturally-occurred environmental waters. Environ. Pollut..

[48] Inam M., Khan R., Park D., Lee Y.-W., Yeom I. (2018). Removal of Sb(III) and Sb(V) by Ferric Chloride Coagulation: Implications of Fe Solubility. Water.

[49] Moussas P.A., Zouboulis A.I. (2008). A study on the properties and coagulation behaviour of modified inorganic polymeric coagulant—Polyferric silicate sulphate (PFSiS). Sep. Purif. Technol..

