# Supplementary material for: The Removal of CuO Nanoparticles from Water by Conventional Treatment C/F/S: The Effect of pH and Natural Organic Matter
Source: Molecules. 2019 Mar 5;24(5):914. doi: 10.3390/molecules24050914 (PMC6429111; doi:10.3390/molecules24050914)
Supplement: Supplementary file 1 [file molecules-24-00914-s001.pdf]

molecules

Supplementary Information

Removal of CuO Nanoparticles from water by Conventional Treatment C/F/S: Effects of pH and Natural Organic Matter

Rizwan Khan[1], Muhammad Ali Inam[1], Du Ri Park[1], Sarfaraz Khan[2], Muhammad Akram[3] and Ick Tae Yeom[1,*]

[1] Graduate School of Water Resources, Sungkyunkwan University (SKKU) 2066, Suwon 16419, Korea; rizwankhan@skku.edu (R.K.); aliinam@skku.edu (M.A.I.); enfl8709@skku.edu (D.R.P.)
[2] Key Laboratory of the Three Gorges Reservoir Region Eco-Environment, State Ministry of Education, Chongqing University, Chongqing 400045, China; Sfk.jadoon@yahoo.com (S.K.)
[3] Shandong Key Laboratory of Water Pollution Control and Resource Reuse, School of Environmental Science and Engineering, Shandong University, Qingdao 266200, China; m.akramsathio@mail.sdu.edu.cn (M.A.)

* Correspondence: yeom@skku.edu; Tel.: +82-31-299-6699

Received: date; Accepted: date; Published: date

2. Materials and Methods

The removal efficiency of CuO NPs was determined according to the following equation.

$$\alpha = \frac{T_i - T_f}{T_i}$$

Where α is the removal efficiency; T_i and T_f is the initial and final turbidity of solution (NTU).

3. Results and Discussions

Table S1. Physicochemical properties of CuO NPs used in the current study.

Parameter	Unit	Value
Density	g/cm^3	6.372
Vendor-reported size	nm	<50
TEM particle size measured (n=20)	nm	92±12
DLS HDD measured in DI water (n=10)	nm	281±27
BET specific surface area measured (n=3)	m^2/g	29 ± 3
pH_{iep}		8.6
Zeta potential in DI water (pH = 7)	(mV)	+21.3 ± 1.6
Purity by ICP-MS	wt %	98.81
Moisture content by TGA	wt %	1.15

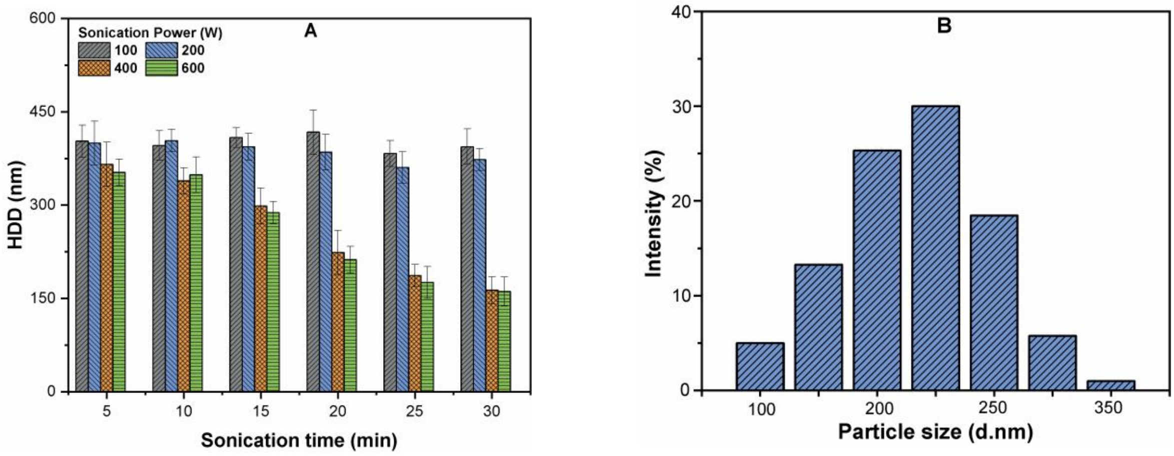

Figure S1. (A) Effects of sonication time (5-30 min) and power (100-600 W) on the dispersion stability of CuO NPs stock (100 mg/L) in DI water; **(B)** Size distribution by the intensity of CuO NPs in DI water after 30 min sonication with ultrasonic power of 400 W;

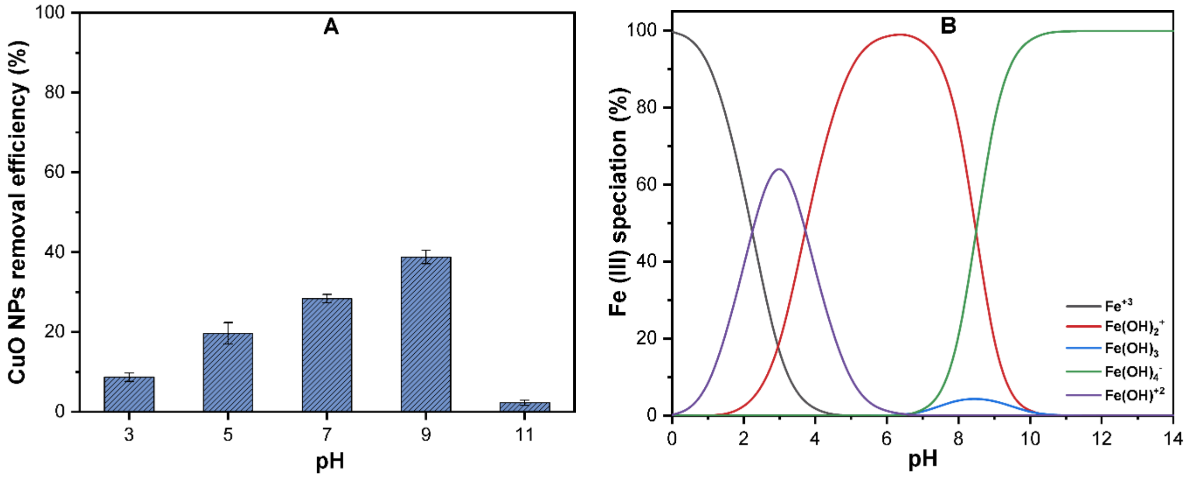

Figure S2. (A) Removal efficiency of CuO NPs (10 mg/L) under control condition at various pH values; **(B)** speciation of Fe(III) as a function of solution pH.

Table.S2. Dissolution of CuO NPs and the change of suspension pH

**Initial solution pH**		**7.0**			**7.0**	
Type of NOM		HA			SA	
NOM Concentration (mg/L)	0	10	20	0	10	20
Solution pH after 24-h	6.90 ± 0.10	6.82 ± 0.05	6.61 ± 0.08	6.90 ± 0.10	6.93 ± 0.07	8.26 ± 0.08
Dissolved Cu^{2+} mg/L	0.701± 0.01	0.912± 0.01	1.787± 0.02	0.701± 0.013	0.819± 0.02	1.140± 0.01
